# Supplementary figures and images for: Neural responses to syllable-induced P1m and social impairment in children with autism spectrum disorder and typically developing Peers
Source: PLoS One. 2024 Mar 8;19(3):e0298020. doi: 10.1371/journal.pone.0298020 (PMC10923473; doi:10.1371/journal.pone.0298020)

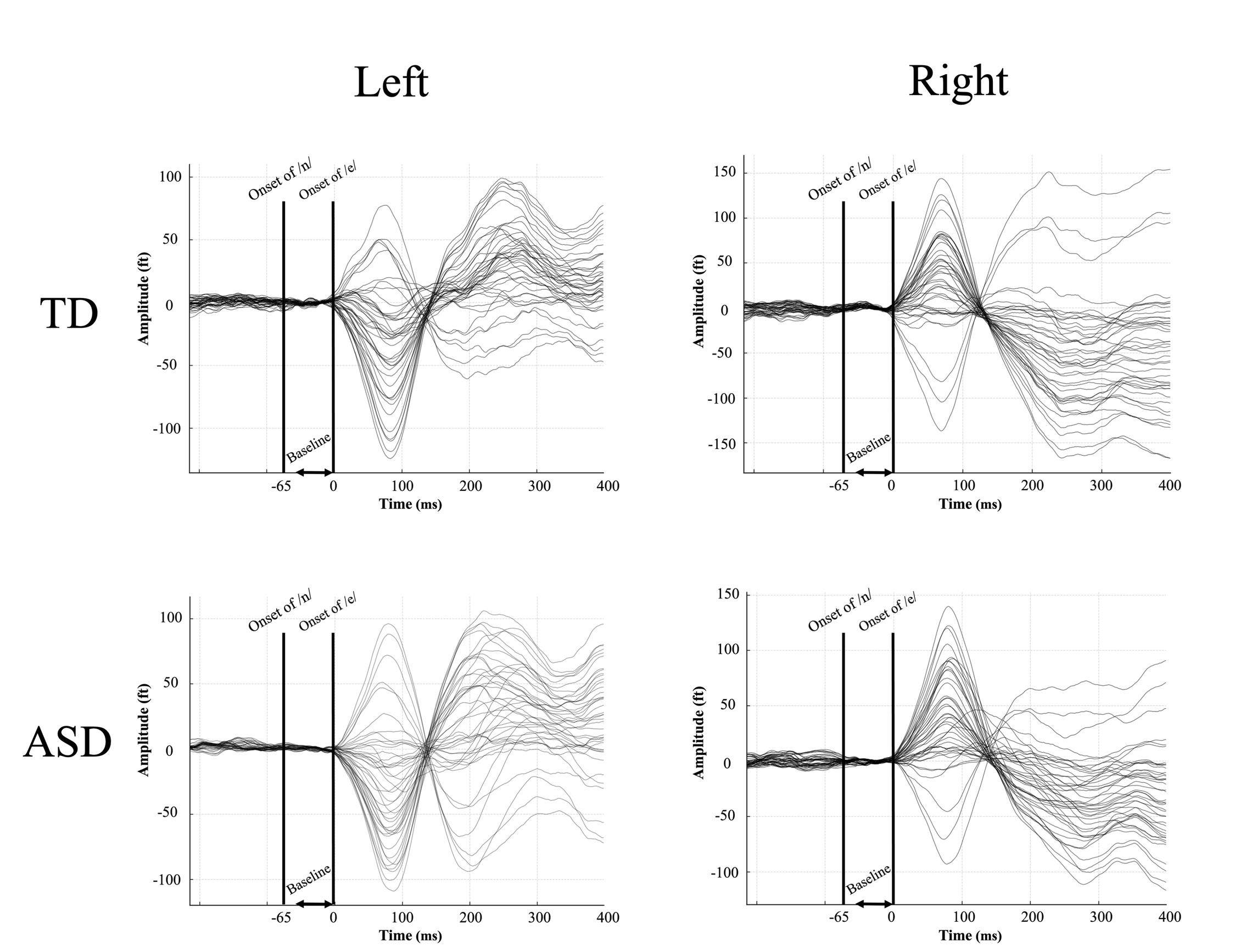

Supplement: S1 Fig — (TIF) [file pone.0298020.s002.tif]
