# Supplementary material for: Neural responses to syllable-induced P1m and social impairment in children with autism spectrum disorder and typically developing Peers
Source: PLoS One. 2024 Mar 8;19(3):e0298020. doi: 10.1371/journal.pone.0298020 (PMC10923473; doi:10.1371/journal.pone.0298020)
Supplement: S1 Table — (PDF) [file pone.0298020.s003.pdf]

Supplementary Table 1. Position of the three coils on the heads of the participants.

	TD(n=24)	ASD (n=40)	t	*p*
**Left Mastoid Process**				
x	71.8 (18.7)	66.5 (7.3)	1.6	0.11
y	11.8 (20.2)	24.4(23.4)	-2.2	0.03*
z	-38.7 (29.4)	-37.0 (27.5)	-0.2	0.82
**Right Mastoid Process**				
x	-69.2 (7.1)	-66.4 (7.9)	-1.41	0.16
y	11.5 (23.9)	25.4 (24.2)	-2.24	0.03*
z	-39.6 (30.2)	-41.7 (27.2)	0.28	0.78
**Nasion**				
x	-0.48 (9.9)	-1.03 (11.7)	0.19	0.85
y	-63.2 (22.5)	-61.0 (20.0)	-0.41	0.68
z	28.7 (21.6)	29.1 (16.3)	-0.08	0.93

TD, typically developing children; ASD, autism spectrum disorder;

*$p < .05$.
