# Supplementary material for: Neural responses to syllable-induced P1m and social impairment in children with autism spectrum disorder and typically developing Peers
Source: PLoS One. 2024 Mar 8;19(3):e0298020. doi: 10.1371/journal.pone.0298020 (PMC10923473; doi:10.1371/journal.pone.0298020)
Supplement: S2 Table — (PDF) [file pone.0298020.s004.pdf]

Supplementary Table 2. Association between SRS-total T-score and right or left P1m log-intensity controlling for Mental processing scale score in K-ABC.

	Coeff.	Robust SE	t	*p*	95%CI		F	Prob > F	R^2
**vs.SRS-total T-score**									
Right P1m log-intensity	-1.48	3.95	-0.38	0.708	-9.41	6.45	21.54	<0.001	0.55
Diagnosis	24.74	15.65	1.58	0.120	-6.69	56.17			
Interaction between right P1m log-intensity and diagnosis	-1.55	5.88	-0.26	0.793	-13.35	10.26			
Mental processing scale score	0.07	0.10	0.66	0.513	-0.14	0.28			
**vs.SRS-total T-score**									
Left P1m log-intensity	-3.47	3.44	-1.01	0.318	-10.36	3.43	21.78	<0.001	0.53
Diagnosis	5.13	12.46	0.41	0.682	-19.84	30.10			
Interaction between left P1m log-intensity and diagnosis	5.78	4.32	1.34	0.187	-2.89	14.44			
Mental processing scale score	0.01	0.12	0.10	0.923	-0.23	0.26			

Coeff., regression coefficient; SE, standard error; CI, confidence interval;
