# Supplementary material for: Neural responses to syllable-induced P1m and social impairment in children with autism spectrum disorder and typically developing Peers
Source: PLoS One. 2024 Mar 8;19(3):e0298020. doi: 10.1371/journal.pone.0298020 (PMC10923473; doi:10.1371/journal.pone.0298020)
Supplement: S3 Table — (PDF) [file pone.0298020.s005.pdf]

Supplementary Table 3. Association between SRS-total T-score and right or left P1m log-intensity for each diagnosis group controlling for Mental processing scale score in K-ABC

	Coeff.	Robust SE	t	*p*	95%CI		F	Prob > F	R^2
**vs.SRS-total T-score**									
**TD**									
Right P1m log-intensity	-0.82	3.94	-0.21	0.837	-9.04	7.39	0.03	0.97	<0.01
Mental processing scale score	-0.01	-0.86	-0.06	0.950	-0.19	0.17			
**ASD**									
Right P1m log-intensity	-3.05	4.46	-0.68	0.499	-12.16	6.06	0.44	0.67	0.03
Mental processing scale score	0.10	0.14	0.70	0.486	-0.19	0.39			
**vs.SRS-total T-score**									
**TD**									
Left P1m log-intensity	-3.44	3.51	-0.98	0.340	-10.81	3.93	0.49	0.62	0.05
Mental processing scale score	0.00	0.11	0.03	0.977	-0.22	0.23			
**ASD**									
Left P1m log-intensity	2.31	2.68	0.86	0.396	-3.14	7.76	0.41	0.67	0.16
Mental processing scale score	0.01	0.15	0.09	0.926	-0.29	0.32			

Coeff., regression coefficient; SE, standard error; CI, confidence interval;

*$p<.05$.
