# Supplementary material for: Neural responses to syllable-induced P1m and social impairment in children with autism spectrum disorder and typically developing Peers
Source: PLoS One. 2024 Mar 8;19(3):e0298020. doi: 10.1371/journal.pone.0298020 (PMC10923473; doi:10.1371/journal.pone.0298020)
Supplement: S4 Table — (PDF) [file pone.0298020.s006.pdf]

Supplementary Table 4. Association between SRS-total T-score and right or left P1m latency controlling for Mental processing scale score in K-ABC and signal noise ratio

	Coeff.	Robust SE	t	*p*	95%CI		F	Prob > F	R^2
**vs.SRS-total T-score**									
Left P1m latency	-0.18	0.69	-2.62	0.011*	-0.32	-0.04	22.21	<0.001	0.55
Diagnosis	13.86	10.21	1.36	0.181	-6.63	34.35			
Interaction between left P1m latency and diagnosis	0.85	0.12	0.70	0.488	-0.16	0.32			
Mental processing scale score	-0.02	0.11	-0.19	0.850	-0.25	0.21			
Square root of the number of averages	-1.38	1.49	-0.92	0.359	-4.36	1.61			

	Coeff.	Robust SE	t	*p*	95%CI		F	Prob > F	R^2
**vs.SRS-total T-score**									
**TD**									
Left P1m latency	-0.16	0.06	-2.61	0.018*	-0.29	-0.31	2.52	0.09	0.23
Mental processing scale score	-0.09	0.13	-0.68	0.508	-0.36	0.18			
Square root of the number of averages	0.76	1.92	0.40	0.695	-3.28	4.80			
**ASD**									
Left P1m latency	-0.10	0.10	-1.07	0.294	-0.30	0.09	0.88	0.46	0.05
Mental processing scale score	-0.03	0.15	-0.23	0.819	-0.33	0.26			
Square root of the number of averages	-2.04	1.97	-1.03	0.308	-6.04	1.97			

Coeff., regression coefficient; SE, standard error; CI, confidence interval;

$^*p<.05$.
