# Supplementary material for: Neural responses to syllable-induced P1m and social impairment in children with autism spectrum disorder and typically developing Peers
Source: PLoS One. 2024 Mar 8;19(3):e0298020. doi: 10.1371/journal.pone.0298020 (PMC10923473; doi:10.1371/journal.pone.0298020)
Supplement: S5 Table — (PDF) [file pone.0298020.s007.pdf]

**Supplementary Table 5.** Correlation between the leftward lateralization in log-transformed P1m intensity and coil positions.

| vs. the leftward lateralization in log-transformed P1m intensity | Coeff. | Robust SE | t | *p* | 95%CI | | F | $R^2$ |
|---|---|---|---|---|---|---|---|---|
| **Left Mastoid Process** | | | | | | | | |
| x | -0.002 | 0.003 | -0.74 | 0.46 | -0.009 | 0.004 | 0.55 | 0.005 |
| y | -0.001 | 0.003 | -0.49 | 0.62 | -0.008 | 0.005 | 0.25 | 0.005 |
| z | 0.003 | 0.002 | 1.16 | 0.25 | -0.002 | 0.008 | 1.35 | 0.026 |
| **Right Mastoid Process** | | | | | | | | |
| x | -0.005 | 0.010 | -0.49 | 0.63 | -0.026 | 0.016 | 0.24 | 0.005 |
| y | 0.000 | 0.003 | -0.01 | 0.99 | -0.006 | 0.006 | 0.00 | <0.001 |
| z | 0.002 | 0.003 | 0.76 | 0.45 | -0.003 | 0.007 | 0.58 | 0.012 |
| **Nasion** | | | | | | | | |
| x | -0.016 | 0.006 | -2.61 | 0.01* | -0.029 | -0.003 | 6.79 | 0.138 |
| y | 0.000 | 0.004 | -0.11 | 0.91 | -0.008 | 0.007 | 0.01 | <0.001 |
| z | 0.003 | 0.004 | 0.63 | 0.53 | -0.006 | 0.012 | 0.39 | 0.008 |

Coeff., regression coefficient; SE, standard error; CI, confidence interval
