# Supplementary material for: Neural responses to syllable-induced P1m and social impairment in children with autism spectrum disorder and typically developing Peers
Source: PLoS One. 2024 Mar 8;19(3):e0298020. doi: 10.1371/journal.pone.0298020 (PMC10923473; doi:10.1371/journal.pone.0298020)
Supplement: S6 Table — (PDF) [file pone.0298020.s008.pdf]

Supplementary Table 6. Association between SRS-total T-score and leftward lateralization in P1m log-intensity controlling for Mental processing scale score in K-ABC and signal noise ratio

	Coeff.	Robust SE	t	*p*	95%CI		F	Prob > F	R^2
**vs.SRS-total T-score**									
Leftward lateralization in log-intensity	-2.77	3.17	-0.87	0.386	-9.16	3.61	18.98	<0.001	0.61
Diagnosis	17.57	2.54	6.91	<0.001*	12.45	22.69			
Interaction between Leftward lateralization in log-intensity and diagnosis	10.50	4.42	2.38	0.02*	1.60	19.40			
Mental processing scale score	0.04	0.11	0.40	0.689	-0.18	0.27			
Square root of the number of averages	-2.00	1.50	-1.33	0.190	-5.02	1.03			
**vs.SRS-total T-score**									
**TD**									
Leftward lateralization in log-intensity	-3.05	3.46	-0.88	0.379	-10.35	4.26	0.78	0.52	0.07
Mental processing scale score	-0.08	0.16	-0.53	0.601	-0.41	0.25			
Square root of the number of averages	1.74	2.13	0.81	0.427	-2.76	6.23			
**ASD**									
Leftward lateralization in log-intensity	8.13	2.89	2.81	0.009*	2.18	14.07	4.17	0.02	0.22
Mental processing scale score	0.32	0.15	0.22	0.827	-0.27	0.33			
Square root of the number of averages	-3.39	2.02	-1.67	0.106	-7.55	0.77			

Coeff., regression coefficient; SE, standard error; CI, confidence interval;

Leftward lateralization in log-intensity is defined as the log-transformed P1m intensity in the left hemisphere minus its counterpart in the right

*p<.05.
