# Supplementary material for: Neural responses to syllable-induced P1m and social impairment in children with autism spectrum disorder and typically developing Peers
Source: PLoS One. 2024 Mar 8;19(3):e0298020. doi: 10.1371/journal.pone.0298020 (PMC10923473; doi:10.1371/journal.pone.0298020)
Supplement: S7 Table — (PDF) [file pone.0298020.s009.pdf]

Supplementary Table 7. Association between SRS-total T-score and leftward lateralization in P1m latency controlling for Mental processing scale score in K-ABC.

	Coeff.	Robust SE	t	*p*	95%CI		F	Prob > F	R^2
**vs.SRS-total T-score**									
Leftward lateralization in latency	-0.16	0.72	-0.23	0.822	-0.16	0.13	20.95	<0.001	0.55
Diagnosis	20.10	2.43	8.28	<0.001*	15.22	25.00			
Interaction between Leftward lateralization in latency and diagnosis	-0.03	0.12	-0.29	0.077	-0.27	0.20			
Mental processing scale score	0.29	0.11	0.26	0.796	-0.20	0.26			
**vs.SRS-total T-score**									
**TD**									
Leftward lateralization in latency	-0.02	0.75	-0.23	0.817	-0.17	0.14	0.04	0.96	<0.01
Mental processing scale score	-0.02	0.11	-0.16	0.871	-0.25	0.22			
**ASD**									
Leftward lateralization in latency	-0.05	0.09	-0.54	0.590	-0.24	0.14	0.18	0.83	0.01
Mental processing scale score	0.05	0.15	0.32	0.753	-0.25	0.35			

Coeff., regression coefficient; SE, standard error; CI, confidence interval;

Leftward lateralization in latency is defined as P1m latency in the left hemisphere minus that in the right

*p<.05.
