# Supplementary material for: Neural responses to syllable-induced P1m and social impairment in children with autism spectrum disorder and typically developing Peers
Source: PLoS One. 2024 Mar 8;19(3):e0298020. doi: 10.1371/journal.pone.0298020 (PMC10923473; doi:10.1371/journal.pone.0298020)
Supplement: S8 Table — (PDF) [file pone.0298020.s010.pdf]

Supplementary Tables 8. All results of analyses with new subjects only.

n-Table 1. Characteristics of participants (new subjects only).

N	ASD *N=28*	TD *N=18*	t or χ^2	*p*
Sex (%boys)	64%	78%	0.942	0.332
Age in months	[illegible]7.3 (8.8)	69.2 (11.9)	0.648	0.521
SRS T-scores				
Total	[illegible]2.9 (11.4)	50.7 (6.9)	-7.407	0.000*
Social awareness	[illegible]7.7 (9.5)	50.1 (7.0)	-6.772	0.000*
Social cognition	[illegible]4.8 (12.7)	51.7 (10.8)	-6.361	0.000*
Social communication	[illegible]9.9 (10.5)	49.7 (6.2)	-7.348	0.000*
Social motivation	[illegible]0.5 (10.2)	53.2 (8.8)	-2.500	0.000*
Autistic mannerism	[illegible]5.9 (16.7)	49.5 (8.5)	-6.181	0.000*
K-ABC Mental processing scale score	[illegible]2.8 (14.7)	[illegible]03.7 (11.1)	0.208	0.836

Numbers are mean (standard deviation) or counts.

K-ABC, Kaufman Assessment Battery for Children;

*p<.05.

n-Table 2. Association between SRS-total T-score and right or left P1m latency controlling for Mental processing scale score in K-ABC (new subjects only).

	Coeff.	Robust SE	t	*p*	95%CI		F	Prob > F	R^2
**vs.SRS-total T-score**									
Right P1m Latency	-0.08	0.11	-0.69	0.498	-0.31	0.16	18.99	<0.001	0.60
Diagnosis	18.39	11.60	1.59	0.123	-5.30	42.08			
Interaction between right P1m latency and diagnosis	0.03	0.16	0.15	0.878	-0.31	0.36			
Mental processing scale score	-0.04	0.11	-0.38	0.708	-0.27	0.19			
**vs.SRS-total T-score**									
Left P1m latency	-0.15	0.10	-1.55	0.131	-0.35	0.05	15.50	<0.001	0.58
Diagnosis	23.08	11.54	2.00	0.054	-0.42	46.58			
Interaction between left P1m latency and diagnosis	-0.04	0.15	-0.25	0.806	-0.34	0.27			
Mental processing scale score	-0.03	0.12	-0.21	0.836	-0.28	0.23			

Coeff., regression coefficient; SE, standard error; CI, confidence interval;

*p<.05.

n-Table 3. Association between SRS-total T-score and right or left P1m latency for each diagnosis group controlling for Mental processing scale score in K-ABC (new subjects only).

	Coeff.	Robust SE	t	*p*	95%CI		F	Prob > F	R^2
**vs.SRS-total T-score**									
**TD**									
Right P1m latency	-0.08	0.12	-0.66	0.519	-0.33	0.18	0.39	0.68	0.07
Mental processing scale score	-0.09	0.12	-0.73	0.479	-0.34	0.17			
**ASD**									
Right P1m latency	-0.05	0.12	-0.45	0.662	-0.30	0.19	0.10	0.91	0.01
Mental processing scale score	-0.01	0.17	-0.09	0.932	-0.37	0.34			
**vs.SRS-total T-score**									
**TD**									
Left P1m latency	-0.14	0.11	-1.34	0.206	-0.38	0.09	1.44	0.28	0.14
Mental processing scale score	-0.06	0.18	-0.35	0.733	-0.45	0.33			
**ASD**									
Left P1m latency	-0.19	0.12	-1.65	0.116	-0.43	0.05	1.36	0.28	0.14
Mental processing scale score	-0.02	0.15	-0.10	0.923	-0.34	0.31			

Coeff., regression coefficient; SE, standard error; CI, confidence interval;

*p<.05.

n-Table 4. Association between SRS-total T-score and leftward lateralization in P1m log-intensity controlling for Mental processing scale score in K-ABC (new subjects only).

	Coeff.	Robust SE	t	*p*	95%CI		F	Prob > F	R^2
**vs.SRS-total T-score**									
Leftward lateralization in log-intensity	-5.02	3.87	-1.30	0.206	-12.97	2.92	19.97	<0.001	0.63
Diagnosis	16.65	2.77	6.00	0.000*	10.96	22.34			
Interaction between Leftward lateralization in log-intensity and diagnosis	12.36	5.43	2.28	0.031*	1.22	23.50			
Mental processing scale score	-0.03	0.11	-0.27	0.788	-0.26	0.20			
**vs.SRS-total T-score**									
**TD**									
Leftward lateralization in log-intensity	-6.03	4.26	-1.41	0.185	-15.40	3.35	1.64	0.24	0.17
Mental processing scale score	-0.20	0.17	-1.19	0.257	-0.58	0.17			
**ASD**									
Leftward lateralization in log-intensity	7.64	3.18	2.40	0.030*	0.86	14.43	6.15	0.01	0.20
Mental processing scale score	0.04	0.14	0.29	0.773	-0.26	0.34			

Coeff., regression coefficient; SE, standard error; CI, confidence interval;

Leftward lateralization in log-intensity is defined as the log-transformed P1m intensity in the left hemisphere minus its counterpart in the right

*p<.05.

n-Supplementary Table 1. Association between SRS-total T-score and right or left P1m log-intensity controlling for Mental processing scale score in K-ABC (new subjects only).

	Coeff.	Robust SE	t	*p*	95%CI		F	Prob > F	R^2
**vs.SRS-total T-score**									
Right P1m log-intensity	0.63	4.11	0.15	0.879	-7.76	9.03	26.41	<0.001	0.62
Diagnosis	40.32	14.96	2.70	0.011	9.77	70.87			
Interaction between right P1m log-intensity and diagnosis	-7.75	5.72	-1.36	0.185	-19.43	3.92			
Mental processing scale score	-0.03	0.11	-0.27	0.790	-0.24	0.19			
**vs.SRS-total T-score**									
Left P1m log-intensity	-2.52	3.83	-0.66	0.515	-10.33	5.28	12.37	<0.001	0.53
Diagnosis	8.67	13.55	0.64	0.527	-18.94	36.27			
Interaction between left P1m log-intensity and diagnosis	3.79	4.78	0.79	0.434	-5.94	13.52			
Mental processing scale score	-0.12	0.15	-0.78	0.439	-0.42	0.19			

Coeff., regression coefficient; SE, standard error; CI, confidence interval;

n-Supplementary Table 2. Association between SRS-total T-score and right or left P1m log-intensity for each diagnosis group controlling for Mental processing scale score in K-ABC (new subjects only).

	Coeff.	Robust SE	t	*p*	95%CI		F	Prob > F	R^2
**vs.SRS-total T-score**									
**TD**									
Right P1m log-intensity	1.50	3.89	0.39	0.706	-6.91	9.91	0.51	0.61	0.03
Mental processing scale score	-0.11	0.12	-0.98	0.346	-0.37	0.14			
**ASD**									
Right P1m log-intensity	-7.28	3.54	-2.06	0.056	-14.79	0.23	2.57	0.11	0.10
Mental processing scale score	0.02	0.15	0.12	0.905	-0.30	0.33			
**vs.SRS-total T-score**									
**TD**									
Left P1m log-intensity	-2.53	4.16	-0.61	0.555	-11.69	6.63	0.67	0.53	0.07
Mental processing scale score	-0.12	0.18	-0.65	0.528	-0.51	0.28			
**ASD**									
Left P1m log-intensity	1.27	3.01	0.42	0.679	-5.02	7.55	0.24	0.79	0.03
Mental processing scale score	-0.12	0.19	-0.63	0.536	-0.51	0.27			

Coeff., regression coefficient; SE, standard error; CI, confidence interval;

*p<.05.

n-Supplementary Table3. Association between SRS-total T-score and right or left P1m latency controlling for Mental processing scale score in K-ABC and signal noise ratio (new subjects only).

	Coeff.	Robust SE	t	*p*	95%CI		F	Prob > F	R^2
**vs.SRS-total T-score**									
Left P1m latency	-0.14	0.09	-1.51	0.141	-0.32	0.05	13.13	<0.001	0.60
Diagnosis	22.23	10.86	2.05	0.049*	0.07	44.39			
Interaction between left P1m latency and diagnosis	-0.02	0.14	-0.11	0.910	-0.29	0.26			
Mental processing scale score	-0.02	0.13	-0.17	0.863	-0.28	0.24			
Square root of the number of averages	1.75	1.58	1.11	0.276	-1.47	4.96			
**vs.SRS-total T-score**									
**TD**									
Left P1m latency	-0.09	0.11	-0.75	0.470	-0.34	0.17	2.69	0.10	0.29
Mental processing scale score	-0.20	0.25	-0.79	0.447	-0.76	0.36			
Square root of the number of averages	3.69	2.83	1.30	0.221	-2.61	10.00			
**ASD**									
Left P1m latency	-0.17	0.10	-1.65	0.116	-0.38	0.05	0.95	0.44	0.16
Mental processing scale score	0.00	0.16	0.02	0.985	-0.33	0.33			
Square root of the number of averages	1.25	2.22	0.56	0.579	-3.39	5.89			

Coeff., regression coefficient; SE, standard error; CI, confidence interval;

*p<.05.

n-Supplementary Table 4. Association between SRS-total T-score and leftward lateralization in P1m log-intensity controlling for Mental processing scale score in K-ABC and signal noise ratio (new subjects only).

	Coeff.	Robust SE	t	*p*	95%CI		F	Prob > F	R^2
**vs.SRS-total T-score**									
Leftward lateralization in log-intensity	-4.64	4.10	-1.13	0.268	-13.07	3.78	16.86	<0.001	0.64
Diagnosis	17.83	2.73	6.52	0.000*	12.21	23.46			
Interaction between Leftward lateralization in log-intensity and diagnosis	11.16	5.84	1.91	0.067	-0.85	23.17			
Mental processing scale score	-0.03	0.12	-0.26	0.796	-0.27	0.21			
Square root of the number of averages	1.33	1.98	0.67	0.508	-2.74	5.39			
**vs.SRS-total T-score**									
**TD**									
Leftward lateralization in log-intensity	-5.52	4.86	-1.13	0.283	-16.36	5.32	5.38	0.02	0.37
Mental processing scale score	-0.31	0.18	-1.70	0.120	-0.72	0.10			
Square root of the number of averages	4.00	2.38	1.68	0.124	-1.31	9.31			
**ASD**									
Leftward lateralization in log-intensity	7.41	3.57	2.08	0.057	-0.24	15.07	3.90	0.03	0.20
Mental processing scale score	0.05	0.14	0.32	0.752	-0.26	0.35			
Square root of the number of averages	0.40	3.12	0.13	0.900	-6.29	7.09			

Coeff., regression coefficient; SE, standard error; CI, confidence interval;

Leftward lateralization in log-intensity is defined as the log-transformed P1m intensity in the left hemisphere minus its counterpart in the right

*p<.05.

n-Supplementary Table 5. Association between SRS-total T-score and leftward lateralization in P1m latency controlling for Mental processing scale score in K-ABC (new subjects only).

	Coeff.	Robust SE	t	*p*	95%CI		F	Prob > F	R^2
**vs.SRS-total T-score**									
Leftward lateralization in latency	-0.01	0.06	-0.14	0.889	-0.14	0.12	19.06	<0.001	0.57
Diagnosis	19.06	2.69	7.08	0.000*	13.54	24.58			
Interaction between Leftward lateralization in latency and diagnosis	-0.06	0.11	-0.54	0.591	-0.29	0.17			
Mental processing scale score	-0.02	0.12	-0.14	0.891	-0.26	0.23			
**vs.SRS-total T-score**									
**TD**									
Leftward lateralization in latency	0.00	0.07	0.05	0.962	-0.16	0.17	0.33	0.73	0.04
Mental processing scale score	-0.14	0.18	-0.77	0.458	-0.54	0.26			
**ASD**									
Leftward lateralization in latency	-0.08	0.09	-0.93	0.365	-0.27	0.10	0.47	0.63	0.03
Mental processing scale score	0.04	0.15	0.26	0.801	-0.27	0.35			

Coeff., regression coefficient; SE, standard error; CI, confidence interval;

Leftward lateralization in latency is defined as P1m latency in the left hemisphere minus that in the right

*p<.05.
