# Supplementary material for: Neural responses to syllable-induced P1m and social impairment in children with autism spectrum disorder and typically developing Peers
Source: PLoS One. 2024 Mar 8;19(3):e0298020. doi: 10.1371/journal.pone.0298020 (PMC10923473; doi:10.1371/journal.pone.0298020)
Supplement: Attachment — Submitted filename: rev.docx [file pone.0298020.s013.docx]

I appreciate the revisions made by the authors. They discovered an error in data processing and reanalyzed the data. In fact, the entire text has changed, including results, discussion, and even the title of the manuscript. However, I still have a few comments and/or suggestions:

**Major**

1. The authors noted that in the ‘limitations’ that ‘… limitation arises from setting thebaseline relative to the onset of the vowel /e/. Brain responses to the preceding consonant /n/ may exist, and the proportion of participants demonstrating this response might differ between the TD and ASD groups, potentially influencing the results. To validate this, future research should include both /ne/ and /e/ stimuli.’

In response to the reviewer #2 they also noted that ‘In the first manuscript, the baseline was mistakenly set to −50 ms to 0 ms relative to the onset of the /n/ consonant’.

This means that in the previous study they analyzed P1m in the time range that overlapped with the range of baselines used in the present study. Why do they believe that the results obtained with a more neutral baseline (i.e., before the presentation of the syllable /ne/) are less correct than the present results where the baseline overlaps with the presentation of the consonant /n/? Arguments and illustrations are needed to support the choice of baseline.

2. The authors included in the introduction a paragraph about ‘P1m and "suppression" phenomenon’. However, they did not investigate the suppression (gating) in their study. Moreover, gating is usually investigated using short non–speech stimuli, such as clicks, while Hirosawa et al used speech stimuli. Therefore, I am not sure that this paragraph is relevant for the discussion.

On the other hand, there is literature that may be more relevant. Indeed, P1 abnormalities were reported in the auditory processing disorder (APD). P1 amplitude evoked by a speech stimulus (/da/) has been shown to be smaller in children with APD compared to TD control children (Sharma et al, 2014; DOI: <http://dx.doi.org/10.1055/s-0033-1363524>). Another recent study also found P1 abnormalities in APD in response to speech stimuli (doi: 10.1016/j.ijporl.2021.110944). As many children with ASD may have APD (James, 2022; https://doi.org/10.1016/j.jcomdis.2022.106252), P1 finding of Hirosawa et al may, at least to some expend, reflect central auditory processing deficit, rather than ASD itself. APD and receptive language were not assessed in the present study. It seems to be an important limitation, which needs to be discussed.

3. In p. 14, the authors report no group differences in P1 amplitude and latency. Does the asymmetry of P1 amplitude differ between TD and ASD? (I apologize if I missed this information. Perhaps it would have been better to report it directly after the group differences in latency and amplitude).

4. Thank you for your response to comment 8. You write: ‘However, this study did not account for the impact of fine head movements and variations in head shape.’

This is not exactly what my concern was about. The reviewer is aware of a case in which the group mean head positions of subjects from two different clinical groups differed statistically significantly in the left-to-right direction during MEG recording. Even if you excluded subjects who moved a lot, the left-right position by itself (not ‘fine head movements’ or ‘head shape’) could differ between ASD and TD participants, which in turn might affect the results. Once you have measured the head position of each subject at least once, you could probably report information about the differences between ASD and TD (left-to-right position, rotation), and also discuss the limitations (not tracking head position).

It would also be interesting to know whether L-R P1m lateralization correlates with this [baseline] position.

5. Fig 1. Waveform of the standard /ne/ (left) and deviant /Ne/ (right) voice stimulus. The total duration of each stimulus was 342 ms, segmented into 65 ms for the consonant /n/ and 277 ms for the subsequent vowel sound /e/. The MEG analysis onset time was defined as the beginning of the vowel portion. It is important to note that only the standard stimuli were used for the subsequent equivalent current dipole (ECD) estimation, as only 328 this condition provided a sufficient number of epochs for accurate ECD calculation.

From this description it is unclear what is presented in figure 1. Is it a representative subject or the group average? Please provide figures for this time interval separately for TD and ASD subjects (each group average) in the left and right hemispheres.

Discussion.

6. The P1m results are interesting to discuss in relation to the possible contribution of the sustained negative shift of current, which has been described in adults ( Gutschalk and Uppenkamp, 2011; doi:10.1016/j.neuroimage.2011.02.026) and, more recently ,in both adults and children (Orekhova et al, 2023; <https://doi.org/10.1016/j.cortex.2023.10.020>). Figure 3 in Orekhova et al. shows that the amplitude and possibly the latency of P1 (=P100m) can be influenced by a sustained negative shift associated with the processing of phonetic features of the speech stimulus. The fact that different physiological processes may contribute to auditory ERP/ERF activity over the P1m time range allows for different interpretations of the P1m results.

7. The correlation between P1m latency and SRS was observed in TD group, but not in the ASD. The authors suggested that ‘.. rather than directly indicating autistic traits, a shorter latency of the left P1m might represent a neural adaptation against these traits’.

However, it is not clear if SRS scale reflects the same underlying physiology in TD and ASD. Indeed, in ASD it may reflect serious deficit, such as a degree of E/I imbalance, while it would be strange to expect presence of such deficit in TD.

It's up to the authors to decide, but in my opinion, their interpretation looks a bit naive.

**Minor**

1. Thank you for your detailed response to my previous comment 2. I think this information may be interesting mot only for the reviewer. Therefore, I suggest to summarize briefly the main results in the text (e.g. ‘the correlation was reproduced even for a smaller sample not included in the previous study’) and to address the reader to the S6.

2. Lines 591-

(1)We identified a notable association between a shorter latency of syllable-induced P1m in the left hemisphere and pronounced autistic traits. Interestingly, this correlation is primarily evident in TD children and appears nonsignificant in children with ASD. (2) At a glance, one might infer that a shorter latency of the left P1m serves as a neural marker for prominent autistic traits overall. (3) If this assumption is accurate, then a shorter latency of the left P1m could potentially be linked with lower conceptual inference skills, an identified facet of autistic symptomatology [62].

It is not clear how (3) follows directly from (1 and 2), since conceptual inference skills may reflect IQ rather than the autism disorder itself. According to this logic, one would expect a correlation between P1 latency and IQ/MPS in both groups.

3. Some tables are very difficult to read because it is difficult to understand what raw corresponds to what, e.g.:

4. Fig. 4. Some information in the legend is redundant (e.g. ‘The horizontal axis showcases the latency of P1m in the left hemisphere, ranging from 40 to 120 ms.’, etc.) This one can see on the plot. On the other hand, there are no individual data points, which were present in the previous version. Please show individual data points in figures 4 and 5 (different marks for ASD and TD children). I would suggest removing bars and marking confidence intervals with lines.

Figures 5. It may be convenient for the reader see directly on the plot what is the ‘leftward lateralization’, e.g.:

_______________________________

R>L	R<L
