# Supplementary material for: Neural responses to syllable-induced P1m and social impairment in children with autism spectrum disorder and typically developing Peers
Source: PLoS One. 2024 Mar 8;19(3):e0298020. doi: 10.1371/journal.pone.0298020 (PMC10923473; doi:10.1371/journal.pone.0298020)
Supplement: Attachment — Submitted filename: response_to_reviewers.docx [file pone.0298020.s014.docx]

I appreciate the revisions made by the authors. They discovered an error in data processing and reanalyzed the data. In fact, the entire text has changed, including results, discussion, and even the title of the manuscript. However, I still have a few comments and/or suggestions:

**Major**

1. The authors noted that in the ‘limitations’ that ‘… limitation arises from setting thebaseline relative to the onset of the vowel /e/. Brain responses to the preceding consonant /n/ may exist, and the proportion of participants demonstrating this response might differ between the TD and ASD groups, potentially influencing the results. To validate this, future research should include both /ne/ and /e/ stimuli.’

**Response**: ‘In the first manuscript, the baseline was mistakenly set to −50 ms to 0 ms relative to the onset of the /n/ consonant’.

This means that in the previous study they analyzed P1m in the time range that overlapped with the range of baselines used in the present study. Why do they believe that the results obtained with a more neutral baseline (i.e., before the presentation of the syllable /ne/) are less correct than the present results where the baseline overlaps with the presentation of the consonant /n/? Arguments and illustrations are needed to support the choice of baseline.

Method Section Revision:

**Response**: Thank you for your insightful comment. Per your suggestion, we have refined our manuscript as follows:

We acknowledge the significance of selecting the appropriate onset time for syllable stimuli, specifically between the consonant /n/ and the vowel /e/. To maintain consistency with our previous studies [29, 56-58, 62], which predominantly examined the vowel /e/ response, we opted for this latter setting in our current study. This choice is pivotal for enabling direct comparisons with prior findings, thereby enriching our understanding of auditory processing in typically developing children and children with ASD. However, we must acknowledge a crucial assumption in this approach: a minimal brain response to the consonant /n/ due to its lower sound intensity compared to the vowel /e/. While this assumption would be reasonable, it may not fully encapsulate the natural auditory processing mechanisms and could inadvertently obscure the brain's response to the consonant /n/. This aspect warrants careful consideration in interpreting our findings.

Discussion Section Revision:

We have also updated the Discussion section to address potential methodological limitations:

Our study's focus on the vowel /e/, setting the baseline relative to its onset, inherently implies a possible oversight of the brain's response to the preceding consonant /n/. This approach, while methodologically sound for our current research objectives, may mask nuances in the brain's processing of the /n/ consonant. This limitation is particularly pertinent when considering the differential responses between TD children and children with ASD. Our findings, thus, should be interpreted with an awareness of this potential masking effect. In light of this, future research endeavors should contemplate including stimuli combinations like /ne/ and /e/ to comprehensively investigate the brain's distinct responses to consonants and vowels. Such explorations would be instrumental in deepening our understanding of auditory processing variations between TD and ASD groups, potentially leading to more nuanced insights into their auditory processing characteristics.

We hope these revisions effectively address your concerns and clarify the rationale behind our methodological choices, thereby enhancing the overall quality and integrity of our research.

2. The authors included in the introduction a paragraph about ‘P1m and "suppression" phenomenon’. However, they did not investigate the suppression (gating) in their study. Moreover, gating is usually investigated using short non–speech stimuli, such as clicks, while Hirosawa et al used speech stimuli. Therefore, I am not sure that this paragraph is relevant for the discussion.

On the other hand, there is literature that may be more relevant. Indeed, P1 abnormalities were reported in the auditory processing disorder (APD). P1 amplitude evoked by a speech stimulus (/da/) has been shown to be smaller in children with APD compared to TD control children (Sharma et al, 2014). Another recent study also found P1 abnormalities in APD in response to speech stimuli (Lunardelo, 2021). As many children with ASD may have APD (James, 2022; https://doi.org/10.1016/j.jcomdis.2022.106252), P1 finding of Hirosawa et al may, at least to some expend, reflect central auditory processing deficit, rather than ASD itself. APD and receptive language were not assessed in the present study. It seems to be an important limitation, which needs to be discussed.

**Response**: Per the reviewer's insightful comment, we have made the following amendments to our manuscript:

We have removed the paragraph in the Introduction that discussed P1m and the suppression phenomenon in the context of psychopathologies. This adjustment aligns the focus of our introduction more closely with the scope of our study.

In the Discussion section, particularly in the Limitations subsection, we have incorporated the reviewer's suggestions as follows:

**Limitations Section Revision:**

Another limitation, as underscored by recent research, concerns the potential intersection of Auditory Processing Disorder (APD) symptoms within the ASD population. Studies by Sharma et al. [1] and Lunardelo et al. [2] revealed P1 amplitude abnormalities in children with APD in response to speech stimuli, specifically the /da/ sound, which is similar to the /ne/ sound utilized in our research. These findings imply that P1 irregularities may not be unique to ASD and could also signify a central auditory processing deficit characteristic of APD. Moreover, the work of James et al. [3] indicates a potentially high incidence of APD symptoms among children with ASD. This overlap suggests that the P1 abnormalities we observed in children with ASD might partially reflect a broader spectrum of auditory processing challenges extending beyond the confines of ASD. The absence of a direct evaluation of APD in our study is a notable oversight. This limitation warrants caution in attributing the P1 abnormalities solely to ASD and suggests the need for future research to disentangle the auditory processing profiles of ASD from those of APD. Undertaking such research would provide a more comprehensive understanding of the auditory processing dynamics in neurodevelopmental disorders.

3. In p. 14, the authors report no group differences in P1 amplitude and latency. Does the asymmetry of P1 amplitude differ between TD and ASD? (I apologize if I missed this information. Perhaps it would have been better to report it directly after the group differences in latency and amplitude).

**Response**: Thank you for your insightful comment regarding the presentation of P1 amplitude asymmetry data in our manuscript. We appreciate your attention to this detail and understand the importance of clearly communicating these findings.

In our original manuscript, we reported the absence of significant group differences in P1 amplitude and latency on page 14. We agree with your suggestion that information about the asymmetry of P1 amplitude is also of interest to readers. However, we chose to detail the asymmetry analysis in a later section (*More pronounced autistic symptoms are associated with stronger leftward lateralization in P1m intensity, exclusively in children with ASD*) due to its methodological complexity, including steps like outlier identification and exclusion.

To address your concern and enhance the clarity of our manuscript, we have now included a brief mention of the P1 amplitude asymmetry results in the section discussing group differences in P1 amplitude and latency. Specifically, we have added the following statement on page 14:

“Besides reporting no significant group differences in P1 latency and log-transformed intensity, we also examined the asymmetry of P1 intensity between the TD and ASD groups. It is important to note that this analysis revealed no significant difference in the asymmetry of P1 amplitude between the two groups. We delve into the details of this analysis, including the calculation method and considerations for outlier exclusion, in a later section of this manuscript.”

This addition aims to provide a concise overview of the P1 amplitude asymmetry findings while directing readers to the section where the analysis is discussed in depth. We believe this revision addresses your concern and improves the manuscript by making this information more accessible while maintaining the logical flow and thoroughness of our analysis.

We hope this modification satisfactorily addresses your comment, and thank you again for your constructive feedback.

4. Thank you for your response to comment 8. You write: ‘However, this study did not account for the impact of fine head movements and variations in head shape.’

This is not exactly what my concern was about. The reviewer is aware of a case in which the group mean head positions of subjects from two different clinical groups differed statistically significantly in the left-to-right direction during MEG recording. Even if you excluded subjects who moved a lot, the left-right position by itself (not ‘fine head movements’ or ‘head shape’) could differ between ASD and TD participants, which in turn might affect the results. Once you have measured the head position of each subject at least once, you could probably report information about the differences between ASD and TD (left-to-right position, rotation), and also discuss the limitations (not tracking head position).

It would also be interesting to know whether L-R P1m lateralization correlates with this [baseline] position.

**Response**: Thank you for your valuable feedback regarding the impact of initial head positions on our P1m results. Based on your comment, we have made significant revisions to our manuscript to address these concerns.

In the Results section, we have included a detailed analysis of the initial head positions of the participants, attaching three coils to each subject's skull at both mastoid processes and the nasion. This allowed us to track their initial head positions accurately. Our analysis revealed significant differences between the ASD and TD groups in the y-coordinate of the coil at both the left and right mastoid processes. These findings, which indicate a more posterior position of the mastoid processes in children with ASD compared to TD children, are now thoroughly reported and discussed.

Furthermore, we investigated the correlation between the leftward lateralization in log-transformed P1m intensity and the initial head positions. A significant correlation was found between the leftward lateralization and the x-coordinate of the coil at the nasion, suggesting that a more left-located coil at the nasion corresponds to a larger leftward lateralization of intensity. This correlation, along with the lack of significant associations in other models, is detailed in Supplementary Table 7.

In the Discussion section, particularly under limitations, we acknowledge that despite efforts to monitor and control head position, the significant differences in initial head positions could potentially influence the results of dipole estimation. We discuss the implications of these findings and the need for future research to more thoroughly consider the impact of fine head movements and variations in head shape.

These revisions aim to comprehensively address your concerns about the role of the initial head position in interpreting our P1m results. We believe that these additions enhance the rigor and clarity of our study, providing a more accurate representation of the potential variables influencing our findings.

Thank you again for your constructive feedback, which has been instrumental in strengthening our manuscript.

Result section revision

To ensure that the initial head positions did not differ statistically between the ASD and TD groups, we attached three coils to each subject's skull, positioned at both mastoid processes and the nasion. Each coil created a magnetic field that enabled us to track their initial head positions. A Student’s t-test revealed significant differences between the ASD and TD groups in the y-coordinate of the coil at both the left mastoid process (t(62) = -2.22, p = 0.03) and the right mastoid process (t(62) = -2.05, p = 0.04), which might affect the results (as discussed in the limitations section). No significant differences were observed in the x and z coordinates of these coils. Similarly, no significant differences were found in any coordinate of the coil at the nasion. Detailed results are presented in Supplementary Table 1.

Results section revision

As significant differences were observed in the initial head position (i.e., y-coordinate of the coils at both the left and right mastoid process), we investigated whether the leftward lateralization in log-transformed P1m intensity correlated with these initial head positions. To this end, we employed simple regression analysis to predict the leftward lateralization in log-transformed P1m intensity based on the x, y, or z coordinates of each coil separately. A significant correlation was found between the leftward lateralization in log-transformed P1m intensity and the x coordinate of the coil at the nasion (t(50) = -2.61, p = 0.01), indicating that a larger leftward lateralization of intensity corresponds to a left-located coil at the nasion. No significant associations were observed in any of the other models. Detailed results are presented in Supplementary Table 5.

Discussion section (limitation) revision

In this study, participants were monitored using a video camera to detect noticeable body movements. An examiner accompanied the children in the shielded room and instructed them to maintain a constant head position throughout the experiment. Instances of pronounced body movement were excluded based on noise detection. Additionally, participants who exhibited significant shifts in head position during the session were excluded due to a reduction in the GOF in the P1m dipole analysis. Despite these measures, we observed significant differences in the initial head positions between the two groups. Specifically, the positions of both the right and left mastoid processes in children with ASD were significantly more posterior compared to those in TD children. This difference could reflect variations in initial head positioning or head shape; either factor could potentially influence the results of dipole estimation. Indeed, while the position of the mastoid process did not affect the leftward lateralization of log-transformed P1m intensity, the x-coordinate of the coil at the nasion was found to significantly influence the estimation of this parameter. Furthermore, this study did not comprehensively account for the impact of fine head movements and variations in head shape, which are factors that could introduce additional variability in the neuroimaging data. Future research should consider these aspects more thoroughly to mitigate their potential effects on data interpretation.

Supplementary Table 1:

Differences in x, y, and z coordinates of the head coils. Larger (smaller) values of the x coordinate correspond to the left (right) direction, respectively. Larger (smaller) values of the y coordinate correspond to the posterior (anterior) direction, respectively. Larger (smaller) values of the z coordinate correspond to the head (foot) direction, respectively.

Supplementary Table 5.

Correlation between leftward lateralization in log-transformed P1m intensity and initial head positions

5. Fig 1. Waveform of the standard /ne/ (left) and deviant /Ne/ (right) voice stimulus. The total duration of each stimulus was 342 ms, segmented into 65 ms for the consonant /n/ and 277 ms for the subsequent vowel sound /e/. The MEG analysis onset time was defined as the beginning of the vowel portion. It is important to note that only the standard stimuli were used for the subsequent equivalent current dipole (ECD) estimation, as only 328 this condition provided a sufficient number of epochs for accurate ECD calculation.

From this description it is unclear what is presented in figure 1. Is it a representative subject or the group average? Please provide figures for this time interval separately for TD and ASD subjects (each group average) in the left and right hemispheres.

**Response**: Thank you for your valuable feedback regarding the visual representation of our data in the manuscript. We understand your interest in seeing the group averages for the TD and ASD participants in both hemispheres.

To clarify, Figure 1 in our manuscript is intended to depict the waveform of the auditory stimuli (standard /ne/ and deviant /Ne/) used in our study. Its purpose is to provide a clear understanding of the stimuli's structure and duration, which is foundational for our experiment. The figure shows the sound waveforms, demonstrating the segmentation of the consonant /n/ and the vowel /e/.

Additionally, we have noted your interest in viewing the MEG response data for both TD and ASD participants. While we originally presented a representative subject's neural response in Figure 2, we acknowledge the value of your suggestion. To address this, we have decided to include new Supplementary Figure 1 that displays the group averages for the TD and ASD participants in the left and right hemispheres. These figures will provide a comprehensive view of the neural response patterns across both groups and hemispheres, adding depth to our analysis and discussion.

We believe these additions, along with the clarification of Figure 1's purpose, will enhance the understanding and impact of our study. We hope this modification meets your requirements and further strengthens our manuscript.

Now legends of Fig. 1 and Fig. 2 read as follows:

Fig 1. Waveform of the Auditory Stimuli.

This figure presents the sound waveforms of the standard /ne/ (left panel) and deviant /Ne/ (right panel) voice stimuli used in the study. The total duration of each stimulus is 342 ms, segmented into 65 ms for the consonant /n/ and 277 ms for the subsequent vowel sound /e/. This illustration is intended to provide a clear understanding of the structural and temporal characteristics of the stimuli employed in our experiments. The MEG analysis onset time was defined as the beginning of the vowel portion. It is important to note that only the standard stimuli were used for the subsequent equivalent current dipole (ECD) estimation, as only this condition provided a sufficient number of epochs for accurate ECD calculation.

Fig 2. Neuromagnetic response to the standard syllable /ne/ stimuli.

This figure presents these waveforms and the magnetic contour map of P1m for a representative participant. Syllable-induced AEF with a baseline from −50 to 0 ms relative to the onset of the vowel /e/. The resultant AEF displayed a pronounced activity peak between 45 and 150 ms. The onset of the consonant /n/ is at −65 ms relative to that of /e/. The blue arrow displays the direction of the estimated dipole moment.

S1_Fig. Neuromagnetic response to the standard syllable /ne/ stimuli for typical development（TD）and autism spectrum disorder（ASD）participants (each group average) in the left and right hemispheres. Syllable-induced AEF with a baseline from −50 to 0 ms relative to the onset of the vowel /e/. The onset of the consonant /n/ is at −65 ms relative to that of /e/.

Discussion.

6. The P1m results are interesting to discuss in relation to the possible contribution of the sustained negative shift of current, which has been described in adults ( Gutschalk and Uppenkamp, 2011; doi:10.1016/j.neuroimage.2011.02.026) and, more recently, in both adults and children (Orekhova et al, 2023; <https://doi.org/10.1016/j.cortex.2023.10.020>). Figure 3 in Orekhova et al. shows that the amplitude and possibly the latency of P1 (=P100m) can be influenced by a sustained negative shift associated with the processing of phonetic features of the speech stimulus. The fact that different physiological processes may contribute to auditory ERP/ERF activity over the P1m time range allows for different interpretations of the P1m results.

**Response**: Thank you for your insightful comment regarding the interpretation of our P1m results, particularly in relation to the potential contribution of the sustained negative shift of current, as described in the studies by Gutschalk and Uppenkamp (2011) and Orekhova et al. (2023). Your observation about the possible influence of this sustained negative shift on both the amplitude and latency of the P1m response is indeed a significant consideration for our study.

In response to your comment, we have revised our manuscript to incorporate a more detailed discussion. We have explored how the sustained field (SF), associated with the processing of periodicity/pitch and formant structure in speech stimuli, might interact with and influence the P1m component. This SF, as evidenced in the literature, is thought to reflect the activity of non-synchronized neuronal populations in the auditory cortex, which serve as feature detectors for complex sounds. The potential for this SF to be present in the time range of the P1m component or even earlier suggests that our observed association between autistic traits and syllable-induced P1m latency might also encompass the processing of these perceptually salient features of speech stimuli.

Additionally, we have considered the implications of these findings for understanding auditory processing in children with ASD. The emerging evidence points to atypical attentional responses to speech and higher-level processing anomalies in this population, which could be intertwined with the dynamics of the sustained negative shift.

Our revised discussion section now offers a richer interpretation of the P1m results, considering the complexity of auditory processing and the potential co-occurrence of early auditory responses with more elaborate phonetic feature processing. We believe this enhanced discussion addresses your concern and adds depth to our analysis, aligning with the evolving understanding of auditory processing mechanisms in neurodevelopmental disorders like ASD.

We appreciate your valuable feedback, which has significantly contributed to the improvement of our manuscript.

**Discussion section revision:**

Another approach to interpreting our P1m results involves considering the broader framework of auditory processing, particularly in relation to the potential contribution of the 'sustained negative shift' of current, as described in adults [4] and in both adults and children [5]. The processing of sounds characterized by periodicity/pitch and/or formant structure, such as vowels, is associated with a greater sustained negative shift of cortical source current, known as the sustained field (SF), which persists throughout stimulus presentation. This SF, captured by MEG/EEG, is thought to reflect the activation of non-synchronized neuronal populations [13,14]. These neurons function as 'feature detectors' for perceptually salient features of complex sounds, facilitating higher-level processing [15,16,17]. The enhancement of MEG/EEG-measured SF occurs when stimuli are perceptually salient [7] or carry semantic meaning [6], and its magnitude varies with phonetic features, such as periodic versus non-periodic vowels [5]. Notably, SF is evident in the time range of the P1m component or even earlier, suggesting that the co-occurrence of SF with P1m might influence the contour and amplitude of the P1m. This interaction is particularly relevant in our findings, where we observed an association between autistic traits and syllable-induced P1m latency and its leftwared lateralization in intensity, possibly reflecting a latent relationship between autistic traits and SF. The potential connection between autistic traits and SF is compelling, given the emerging behavioral and electrophysiological evidence of impaired attentional responses to speech in children with ASD. This might imply reduced perceptual salience of speech stimuli and atypical higher-level processing. Earlier studies indicate that children with ASD exhibit specific deficits in orienting to vowel sounds compared to simple and complex tones [8], highlighting their potentially reduced perceptual salience to speech. Moreover, these deficits may be linked to atypical higher-level processing of auditory stimuli in this population [9]. Given these considerations, future research aiming to further elucidate the complex interplay between autistic traits, SF, and properties of P1m could offer a richer understanding of the neural basis of ASD and how it is reflected in MEG/EEG measurements.

7. The correlation between P1m latency and SRS was observed in TD group, but not in the ASD. The authors suggested that ‘.. rather than directly indicating autistic traits, a shorter latency of the left P1m might represent a neural adaptation against these traits’.

However, it is not clear if SRS scale reflects the same underlying physiology in TD and ASD. Indeed, in ASD it may reflect serious deficit, such as a degree of E/I imbalance, while it would be strange to expect presence of such deficit in TD. It's up to the authors to decide, but in my opinion, their interpretation looks a bit naive.

**Response**: Thank you for your insightful comments regarding the interpretation of the correlation between P1m latency and SRS scores in our study. We appreciate your observation that the SRS scale may not reflect the same underlying physiological phenomena in TD and ASD individuals. In response to your feedback, we have revised our manuscript to offer a more nuanced interpretation of these findings.

In the revised section, we acknowledge that the SRS in TD children could represent a range of cognitive processing styles related to autistic traits, while in ASD children, it might reflect more specific aspects of ASD pathology, such as excitatory/inhibitory (E/I) imbalances. This distinction is important to consider, as it suggests that the shorter P1m latency observed in TD children could be indicative of a broad spectrum of cognitive processing, not necessarily direct markers of autistic pathology. Conversely, the lack of a significant correlation in ASD children suggests the involvement of different neural processes, potentially linked to specific neurophysiological characteristics characteristic of ASD.

We also address the possibility that the shorter P1m latency in TD children might reflect neural processes less directly related to autistic pathology, perhaps involved in more general social information processing. This interpretation contrasts with the situation in children with ASD, where the pronounced social deficits might be more directly related to autism-specific pathology, which might not be reflected in P1m latency.

Our revision aims to provide a more comprehensive understanding of the complex interplay between auditory processing and social responsiveness traits in both populations. We believe this refined interpretation aligns better with the complexities of neurodevelopmental differences between TD and ASD individuals.

We hope this revised interpretation addresses your concerns and provides a more accurate reflection of the nuanced relationships between neural markers, SRS scores, and the distinct neurodevelopmental contexts of TD and ASD.

Discussion revision:

We identified a significant association between a shorter latency of syllable-induced P1m in the left hemisphere and pronounced autistic traits. Interestingly, this correlation is primarily evident in TD children and appears nonsignificant in children with ASD. At first glance, this finding suggests a potential link between neural auditory processing and autistic traits. However, it is crucial to consider that the SRS scale may not reflect the same underlying physiology in TD and ASD individuals. In TD children, the SRS could represent a range of cognitive processing styles related to autistic traits, while in ASD children, it might reflect more specific aspects of ASD pathology, such as excitatory/inhibitory (E/I) imbalances [18].

From this perspective, the observed correlation between shorter P1m latency and higher SRS scores in TD children could indicate a relationship between P1m latency and a broad spectrum of cognitive processing that relates to autistic traits rather than being solely indicative of autism-specific pathology. This spectrum might include neural adaptations or processing efficiencies unrelated to autism but still captured by SRS scores.

Conversely, the lack of a significant correlation in ASD children hints at the involvement of different neural processes that are reflected in their SRS scores. These processes could be linked to specific neurophysiological characteristics, such as E/I imbalances, which are considered characteristic of ASD pathology [18].

Therefore, the shorter P1m latency in TD children might reflect a neural process less directly related to autistic pathology, perhaps involved in more general social information processing. In contrast, in children with ASD, given their pronounced social deficits, such general social information processing might no longer be associated with the severity of their social deficits. Instead, their social deficits might be more directly related to autism-specific pathology, which might not be reflected in P1m latency. This could explain why we failed to find a significant relation between P1m latency and SRS scores in this population.

Our findings highlight the need for further research to explore the specific neurophysiological underpinnings of SRS scores in both TD and ASD individuals. Future studies should aim to disentangle the relationships between neural markers like P1m latency, autism-specific pathology, and other mechanisms involved in social information processing. Such research would offer a clearer understanding of the complex interplay between auditory processing and social responsiveness traits in both populations.

**Minor**

1. Thank you for your detailed response to my previous comment 2. I think this information may be interesting mot only for the reviewer. Therefore, I suggest to summarize briefly the main results in the text (e.g. ‘the correlation was reproduced even for a smaller sample not included in the previous study’) and to address the reader to the S6.

**Response**: Thank you for your appropriate advice. Following your advice, we have added the following to the Discussion section.

Discussion Section Revision:

We have added the following part at the end of the Discussion section:

**Results of analyses with new participants only**

In the present study, some participants overlapped with participants included in our previous studies. We excluded them and performed all analyses on 'new' participants only to test whether the results could be reproduced. Twenty-eight children with ASD and 18 TD children were included in these analyses. To summarise the main results, the relationship between left P1m latency and SRS T-scores in the TD group did not maintain statistical significance; the association between SRS total T-score and leftward lateralization in P1m log-intensity in the ASD group remained statistically significant. Other detailed results are given in the S8 tables.

2. Lines 591-

(1)We identified a notable association between a shorter latency of syllable-induced P1m in the left hemisphere and pronounced autistic traits. Interestingly, this correlation is primarily evident in TD children and appears nonsignificant in children with ASD. (2) At a glance, one might infer that a shorter latency of the left P1m serves as a neural marker for prominent autistic traits overall. (3) If this assumption is accurate, then a shorter latency of the left P1m could potentially be linked with lower conceptual inference skills, an identified facet of autistic symptomatology [62].

It is not clear how (3) follows directly from (1 and 2), since conceptual inference skills may reflect IQ rather than the autism disorder itself. According to this logic, one would expect a correlation between P1 latency and IQ/MPS in both groups.

**Response**: We have removed that section of the manuscript as it was entirely restructured in response to major comment 7.

3. Some tables are very difficult to read because it is difficult to understand what raw corresponds to what, e.g.:

**Response**: We apologize for the error. We will ensure that such errors do not occur during this submission.

4. Fig. 4. Some information in the legend is redundant (e.g. ‘The horizontal axis showcases the latency of P1m in the left hemisphere, ranging from 40 to 120 ms.’, etc.) This one can see on the plot. On the other hand, there are no individual data points, which were present in the previous version. Please show individual data points in figures 4 and 5 (different marks for ASD and TD children). I would suggest removing bars and marking confidence intervals with lines.

Figures 5. It may be convenient for the reader see directly on the plot what is the ‘leftward lateralization’, e.g.:

_______________________________

R>L	R<L

**Response**: Thank you for your valuable feedback regarding Figures 4 and 5 in our manuscript. We appreciate your suggestions for improving the clarity and informativeness of these figures.

Regarding your comment on Figure 4, we have revised the legend to eliminate redundant information. As you rightly pointed out, certain details, such as the range of P1m latency, are directly observable from the plot and thus do not need to be reiterated in the legend. Additionally, in response to your suggestion, we have now included individual data points in the figure. These points provide a more detailed and informative visualization.

For Figure 5, we have taken steps to directly illustrate the concept of 'leftward lateralization' in the plot itself, making it more accessible and understandable for readers. This change should facilitate a more immediate comprehension of the relationship between SRS total T-scores and P1m's leftward lateralization in log-transformed intensity. We have also ensured that individual data points for both ASD and TD children are clearly represented, with different markers for each group.

We have replaced the bars with lines to mark the confidence intervals, in both figures. This modification not only enhances the visual appeal of the figures but also aids in better interpretation of the confidence intervals.

We believe these changes address your concerns effectively and enhance the overall quality and clarity of the figures. We hope that these revisions meet your approval and improve the manuscript's ability to communicate the results clearly to its readers.

Figure 4: Relationship Between SRS Total T-Scores and P1m Latency in the Left Hemisphere

To visualize the relation between SRS total T-scores and P1m latency in the left hemisphere for TD children, we performed a simple regression to predict SRS total T-scores based solely on P1m latency, excluding the mental processing scale for clarity. The effect of P1m latency on SRS total T-scores remains significant in this simplified model (t(19) = -2.68, p = 0.015). The figure depicts individual data points for TD children. The solid line represents the predicted regression line, and the shaded area around it denotes the 95% confidence intervals based on our regression model.

SRS, social responsiveness scale; TD children, typically developing children.

Figure 5: Relationship Between SRS Total T-Scores and P1m's Leftward Lateralization

This figure illustrates the relationship between SRS total T-scores and leftward lateralization in log-transformed intensity (defined as the log-transformed P1m intensity in the left hemisphere minus that in the right) for both ASD and TD children. Separate simple regressions were performed for each group to predict SRS total T-scores based on this measure of P1m's leftward lateralization, excluding the mental processing scale for clarity. The effect of leftward lateralization in log-transformed P1m intensity on SRS total T-scores was found to be significant only for TD children (t(28) = 2.15, p = 0.04). Individual data points for each group are plotted, with each line corresponding to a diagnostic group, illustrating how predicted SRS scores vary with P1m’s leftward lateralization.

SRS, social responsiveness scale; ASD, autism spectrum disorder; TD children, typically developing children.
